# Recurrent hypofibrinogenemia after reduced-dose tocilizumab rechallenge in rheumatoid arthritis: a case report

**DOI:** 10.3389/fphar.2026.1981054

**Published:** 2026-10-19

**Authors:** Yanbiao Li, Yuhe Yan, Hua Zhou, Eyan Fu, Mengyi Zheng

**Affiliations:** 1 Department of Rheumatology and Immunologys, Quzhou Second People’s Hospital, Quzhou, Zhejiang, China; 2 Graduate School, Shanxi Medical University, Taiyuan, Shanxi, China

**Keywords:** adverse drug reaction (ADR), hypofibrinogenemia, rechallenge, rheumatoid arthritis, tocilizumab

## Abstract

Tocilizumab (TCZ), a monoclonal antibody targeting the interleukin-6 receptor, is widely used for the treatment of rheumatoid arthritis (RA) and other inflammatory diseases. Reductions in fibrinogen levels have been reported during TCZ treatment, and some patients develop hypofibrinogenemia. Clinical experience with TCZ re-exposure after such an event, particularly at a reduced dose, remains limited. We report a 76-year-old woman with RA whose disease remained inadequately controlled despite multiple antirheumatic therapies. On 24 April 2026, intravenous TCZ was reinitiated at 400 mg, with a pretreatment fibrinogen level of 3.29 g/L. Approximately 1 month later, fibrinogen had fallen to 0.85 g/L without clinically apparent bleeding. Further TCZ treatment was withheld, and 20 units of cryoprecipitate were administered. Fibrinogen initially increased to 1.99 g/L and subsequently returned to 2.07 g/L during the period without further TCZ exposure. Her RA subsequently became highly active again and remained inadequately controlled despite ongoing conventional antirheumatic treatment. After discussion of the previous adverse event and the potential risks of re-exposure, TCZ was restarted on 19 July 2026, at a reduced intravenous dose of 240 mg; the most recent fibrinogen level before re-exposure was 2.07 g/L. Her joint symptoms and inflammatory markers improved, but 17 days later fibrinogen again decreased to 1.05 g/L, without clinically evident bleeding. The temporal association with TCZ reinitiation in 2026, subsequent recovery during TCZ withdrawal, and recurrence after reduced-dose rechallenge support a probable association between TCZ and hypofibrinogenemia in this patient. This case shows that hypofibrinogenemia can recur despite substantial TCZ dose reduction in a patient with a previous TCZ-associated episode. If TCZ re-exposure is considered clinically necessary, closer monitoring of fibrinogen may be warranted.

## 1 Introduction

Interleukin-6 (IL-6) plays an important role in the persistent inflammation and disease progression of rheumatoid arthritis (RA) and has become a major therapeutic target in RA. Tocilizumab (TCZ) is a monoclonal antibody directed against the IL-6 receptor. Randomized controlled trials have shown that intravenous TCZ effectively reduces disease activity in patients with moderate-to-severe RA who remain active despite methotrexate treatment (Smolen et al., 2008). Clinical benefit has also been demonstrated in patients with an inadequate response to tumor necrosis factor inhibitors (Emery et al., 2008). These findings have established TCZ as an important treatment option for patients with refractory RA or disease that remains inadequately controlled after multiple lines of therapy.

The role of IL-6 is not limited to inflammation; it also has the effect of promoting fibrinogen production. *In vitro* studies have shown that IL-6 induces an acute-phase response in human hepatocytes, including increased synthesis and secretion of fibrinogen and C-reactive protein (Castell et al., 1989). A reduction in fibrinogen after IL-6 receptor blockade has also been observed in patients with RA (McInnes et al., 2015). Cases of TCZ-associated hypofibrinogenemia were subsequently reported in case series and clinical studies (Martis et al., 2017), and more recent real-world data suggest that hypofibrinogenemia during TCZ treatment may not be uncommon, although its severity, clinical presentation, and management vary considerably between patients (An et al., 2024; Yıldırım et al., 2024). Recurrent hypofibrinogenemia after TCZ re-exposure has been reported, but it remains unclear whether reducing the TCZ dose can mitigate the risk of recurrence. We report a patient with RA who developed marked hypofibrinogenemia after TCZ was reinitiated in 2026 and experienced recurrence after deliberate rechallenge at a substantially reduced dose.

## 2 Case presentation

A 76-year-old woman weighing 56 kg had a 13-year history of seropositive rheumatoid arthritis (RA), with a rheumatoid factor level of 179 IU/mL and an anti-cyclic citrullinated peptide antibody level of 466.03 RU/mL. Detailed documentation of joint involvement at the initial diagnosis was unavailable. During subsequent follow-up, she developed recurrent polyarticular pain and swelling involving the metacarpophalangeal, wrist, and shoulder joints, with progressive deformities of the metacarpophalangeal joints. She also had coexisting interstitial lung disease, and methotrexate had therefore never been used. Infliximab was initiated in June 2016 at 3 mg/kg (150 mg per infusion) and was discontinued in October 2016 after the development of cervical lymph-node tuberculosis. She was subsequently treated mainly with Tripterygium wilfordii and glucocorticoids. Iguratimod was discontinued in 2023 because of a generalized pruritic rash. Intravenous TCZ 400 mg every 4 weeks was first introduced in April 2024 and continued for approximately 6 months, during which no apparent coagulation abnormality was documented; treatment was discontinued in November 2024 for financial reasons. In April 2026, she presented again with worsening polyarticular pain and swelling. Her fibrinogen level was 3.29 g/L on April 20. Review of previous laboratory records showed fibrinogen concentrations ranging from 2.21 to 4.36 g/L on several measurements in 2024, without a pattern of persistent hypofibrinogenemia. After clinical assessment, intravenous TCZ was reinitiated at 400 mg on 24 April 2026.

Her joint symptoms and inflammatory activity improved after TCZ treatment. On May 26, when she returned for a planned subsequent TCZ infusion, routine laboratory testing showed a fibrinogen level of 0.85 g/L. She had no ecchymosis, epistaxis, gingival bleeding, hematuria, melena, or other clinically apparent bleeding. Further TCZ treatment was withheld, and she received 20 units of cryoprecipitate. Fibrinogen increased from 0.85 to 1.99 g/L by May 28. Without further TCZ exposure, subsequent levels were 1.49, 1.85, and 2.07 g/L on June 25, July 10, and July 13, respectively. No clinical bleeding event was observed during this period.

After TCZ was withheld, her RA became active again, with worsening pain and swelling involving the metacarpophalangeal, wrist, and shoulder joints. Her DAS28 score was 5.8. Treatment with prednisone, iguratimod, and Tripterygium wilfordii did not provide satisfactory disease control. Given the persistent high disease activity, the marked clinical response to the previous TCZ infusion, and the patient’s preference, TCZ rechallenge was considered after reassessment of the potential benefits and risks. Following discussion with the patient and her family regarding the previous episode of hypofibrinogenemia and the risk of recurrence, intravenous TCZ was restarted at a reduced dose of 240 mg on 19 July 2026. At the time of rechallenge, concomitant therapy consisted of Tripterygium wilfordii 10 mg three times daily, iguratimod 25 mg twice daily, and prednisone 10 mg once daily. The most recent fibrinogen level before re-exposure was 2.07 g/L. Her joint symptoms subsequently improved, accompanied by a reduction in inflammatory markers: the erythrocyte sedimentation rate decreased from 34 mm/h on July 13 to 10 mm/h on August 5, while C-reactive protein decreased from 9.05 to 1.21 mg/L over the same period.

On August 5, 17 days after TCZ re-exposure, fibrinogen had again decreased to 1.05 g/L. At the same time, prothrombin time was 11.8 s, international normalized ratio 0.97, activated partial thromboplastin time 21.6 s, thrombin time 25.5 s, and D-dimer 3,626 μg/L FEU. No clinically apparent bleeding was observed. At the first fibrinogen nadir on May 26, AST was 24 U/L, ALT 14 U/L, albumin 31.3 g/L, total bilirubin 11.8 μmol/L, ALP 47 U/L, and GGT 10 U/L. At the second nadir on August 5, AST and ALT were 29 and 19 U/L, respectively. Intermittent thrombocytopenia had been documented before TCZ reinitiation in April 2026 and persisted thereafter. Platelet counts were 60 × 10^9/L on July 13 and 58 × 10^9/L on July 19, decreasing to 50 × 10^9/L on August 5. No splenomegaly or obvious abnormality on peripheral blood smear was identified, and the patient was not receiving antiplatelet or anticoagulant therapy. Bone marrow examination was not performed because of thrombocytopenia and the associated bleeding risk. In view of the recurrent decrease in fibrinogen after reduced-dose rechallenge, further TCZ treatment was withheld. No cryoprecipitate was administered after the second episode, and no clinically apparent bleeding occurred. Fibrinogen was 1.28 g/L on August 20 and subsequently increased to 2.45 g/L on September 21 without fibrinogen replacement. The longitudinal changes in fibrinogen in relation to TCZ exposure and major interventions are shown in Figure 1, and key clinical and laboratory findings are summarized in Table 1. Causality assessment using the Naranjo algorithm and the WHO–UMC system supported a probable association between TCZ and hypofibrinogenemia (Table 2).

**FIGURE 1:**
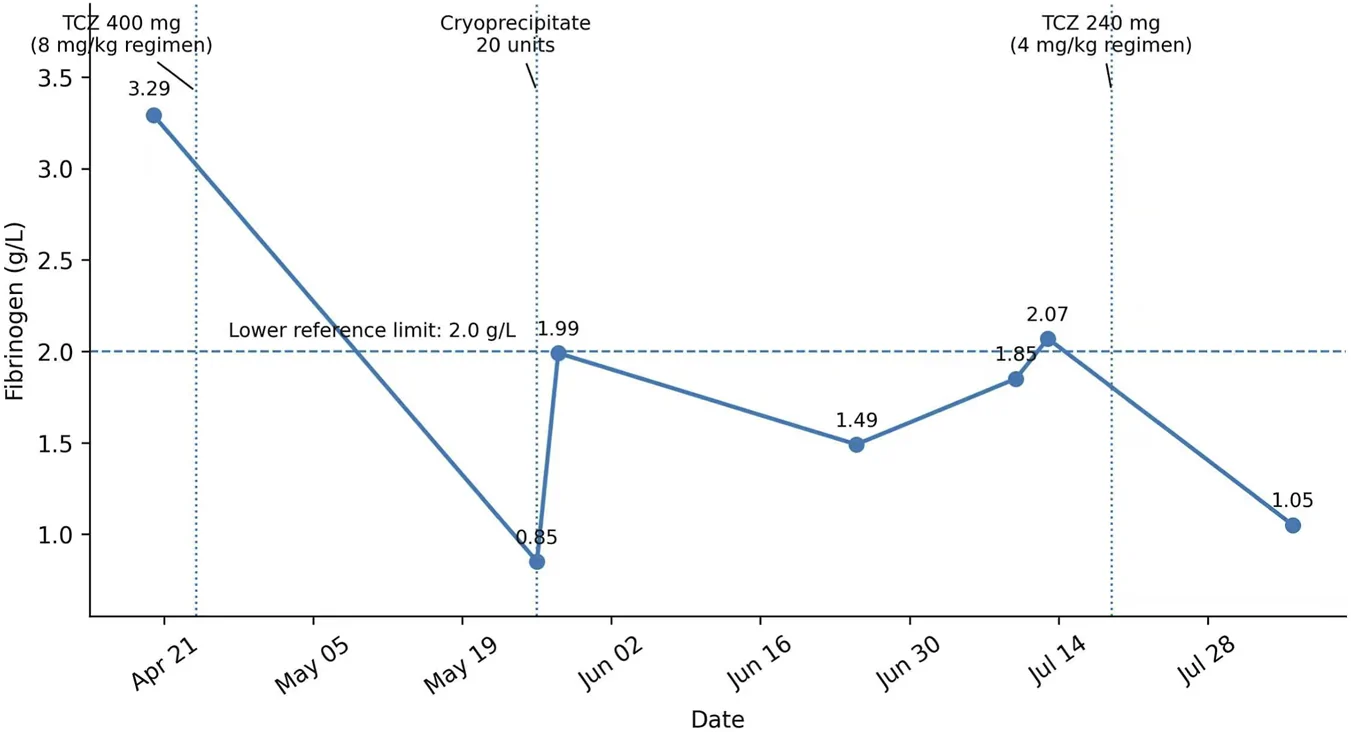
Longitudinal changes in fibrinogen in relation to tocilizumab exposure and major interventions. Fibrinogen levels are shown before and after TCZ reinitiation in 2026 and reduced-dose rechallenge. The dashed horizontal line indicates the lower reference limit for fibrinogen (2.0 g/L).

**TABLE 1:** Key clinical and laboratory findings during the clinical course.

Date	Clinical context	Fibrinogen (g/L)	PLT (×10^9^/L)	PT (s)	TT (s)	CRP (mg/L)	ESR (mm/h)
		Reference range: 2.0–4.0	Reference range: 100–350	Reference range: 10–14	Reference range: 14–21	Reference range: 0–5	Reference range: 0–20
20-Apr-26	Before TCZ reinitiation; TCZ 400 mg given on April 24	3.29	159	11.8	20.4	56.89	54
26-May-26	First hypofibrinogenemia; further TCZ withheld	0.85	77	12.3	26.8	0.56	7
28-May-26	After 20 U cryoprecipitate	1.99	—	11.6	20	—	—
25-Jun-26	Follow-up; RA activity increased	1.49	80	11.7	22.8	1.71	12
13-Jul-26	Before TCZ rechallenge	2.07	60	11	19.7	9.05	34
19-Jul-26	TCZ rechallenge, 240 mg	—	58	—	—	—	—

**TABLE 2:** Causality assessment for tocilizumab-associated hypofibrinogenemia.

Naranjo criterion	Assessment	Score
Previous reports of this reaction	Yes	1
Event occurred after TCZ administration	Yes	2
Improvement afte TCZ withdrawal	Yes*	1
Recurrence after TCZ rechallenge	Yes	2
Alternative causes	Uncertain	0
Reaction after placebo	Not assessed	0
Toxic drug concentration	Not assessed	0
Dose–response relationship	Not established	0
Similar reaction during previous TCZ exposure	No/unknown	0
Objective evidence	Yes	1
Total score	Probable	7
WHO–UMC	Temporal relationship, dechallenge and rechallenge	Probable/Likely

* The first dechallenge was confounded by cryoprecipitate administration. After the second episode, TCZ, was withheld without fibrinogen replacement, and fibrinogen subsequently increased from 1.05 g/L to 1.28 g/L and then to 2.45 g/L, supporting a positive dechallenge.

## 3 Discussion

The key feature of this case was recurrence of hypofibrinogenemia after TCZ was deliberately reintroduced at a substantially reduced dose. Fibrinogen decreased from 3.29 to 0.85 g/L after TCZ was reinitiated at 400 mg in April 2026 and again decreased from 2.07 to 1.05 g/L after rechallenge with 240 mg. Recurrent hypofibrinogenemia after TCZ treatment has also been described previously. Cansu et al. reported a patient with giant cell arteritis whose fibrinogen decreased from 5.06 g/L to 1.80 g/L after the first intravenous TCZ dose of 8 mg/kg. Approximately 6 months later, TCZ was given again because of recurrent disease activity, and fibrinogen decreased again during the second week, although the dose of the second TCZ administration was not clearly reported (Üsküd et al., 2019). Vitiello et al. also described recurrent hypofibrinogenemia after intravenous TCZ, with a similar change occurring again after subsequent subcutaneous treatment (Vitiello et al., 2017). In our patient, fibrinogen again decreased despite reducing the TCZ dose. These reports support the reproducibility of TCZ-associated fibrinogen reduction after re-exposure. The additional feature in our patient was recurrence despite a substantial reduction in the TCZ dose.

The reduction in fibrinogen is pharmacologically plausible because IL-6 is an important regulator of the hepatic acute-phase response. TCZ is a humanized monoclonal antibody against the IL-6 receptor, while fibrinogen is an important acute-phase protein. In the randomized MEASURE study, fibrinogen decreased by about 47% within 4 weeks after TCZ treatment in patients with RA (McInnes et al., 2015). Okano et al. subsequently found that 19 of 65 patients with RA had fibrinogen levels below 2 g/L at 4 weeks after TCZ treatment (Okano et al., 2016). Experimental studies provide a possible explanation for these clinical observations. IL-6 has been shown to promote fibrinogen synthesis and secretion by human hepatocytes in a time- and concentration-dependent manner (Castell et al., 1989; Castell et al., 1990). Further studies have shown that IL-6-related signaling, including STAT3 activation, is involved in the transcriptional regulation of fibrinogen genes (Fuller and Zhang, 2001; Hou et al., 2007). TCZ may therefore reduce IL-6-driven hepatic fibrinogen synthesis through IL-6R blockade, leading to a fall in fibrinogen as inflammation is suppressed. The extent of this effect may vary between patients, and in some patients the decrease may be sufficient to result in hypofibrinogenemia.

From a pharmacological perspective, the fibrinogen changes after the two TCZ exposures are of particular interest. Fibrinogen decreased by approximately 74% after the 400 mg TCZ reinitiation and by approximately 49% after rechallenge with the reduced 240-mg dose. TCZ exhibits concentration-dependent, nonlinear pharmacokinetics. A population pharmacokinetic analysis of 1,793 patients with RA showed that TCZ is eliminated through both linear and nonlinear, target-mediated clearance, with the nonlinear pathway becoming increasingly saturated at higher concentrations (Frey et al., 2010). Consistent with this pharmacokinetic profile, systemic exposure is substantially greater with an 8 mg/kg regimen than with a 4 mg/kg regimen (Zhang and Peck, 2011). The nonlinear pharmacokinetic profile of TCZ may help explain differences in systemic exposure between the two doses but does not account for the timing of fibrinogen decline in this individual patient. Despite the lower dose used for rechallenge in our patient, joint symptoms and inflammatory markers improved, indicating that TCZ retained a clear anti-inflammatory effect, while fibrinogen again decreased. The smaller relative decline during the second exposure was most likely related to the lower TCZ dose and the resulting lower systemic exposure. However, the two treatment periods also differed in baseline fibrinogen concentration, inflammatory activity, and timing of laboratory assessment. In addition, clinical studies have shown considerable interindividual variability in fibrinogen responses to TCZ, with baseline fibrinogen and other patient-related factors potentially influencing the risk of hypofibrinogenemia (An et al., 2024; Wang et al., 2026). Thus, although a dose-related effect appears likely in this patient, other patient- and treatment-related factors may also have contributed. Importantly, dose reduction did not prevent recurrence of hypofibrinogenemia.

However, the causal relationship should be interpreted cautiously. Retrospective application of the ISTH overt DIC scoring system yielded scores of 5 on May 26 and 4 on August 5. The May 26 score reached the numerical threshold for overt DIC, whereas the August 5 score did not. Notably, D-dimer elevation and thrombocytopenia had also been documented outside the hypofibrinogenemic episodes, and the prothrombin time remained within the reference range during both episodes. Consumptive coagulopathy therefore could not be completely excluded. Intermittent thrombocytopenia had been present before TCZ reinitiation and was not accompanied by a parallel progressive decline in hemoglobin or white blood cell counts. After the second episode, fibrinogen increased from 1.05 to 2.45 g/L after TCZ withdrawal without fibrinogen replacement. In this context, the temporal relationship with TCZ exposure and, particularly, recurrence after rechallenge support a probable rather than definitive causal association.

This case indicates that hypofibrinogenemia can recur despite substantial TCZ dose reduction after a previous TCZ-associated episode. When rechallenge is considered clinically necessary, the previous fibrinogen response should be incorporated into the benefit–risk assessment, with close fibrinogen monitoring before and after treatment. Direct comparison of the two episodes is limited by differences in baseline fibrinogen concentration, inflammatory activity, and sampling intervals. In addition, cryoprecipitate administration during the first episode precluded assessment of the natural fibrinogen recovery after initial TCZ withdrawal.

## Data Availability

The original contributions presented in the study are included in the article/supplementary material, further inquiries can be directed to the corresponding author.
